# Inspiratory muscle strength training to improve cardiometabolic health in patients with type 2 diabetes mellitus: protocol for the diabetes inspiratory training clinical trial

**DOI:** 10.3389/fendo.2024.1383131

**Published:** 2024-09-13

**Authors:** Baylee L. Reed, Dallin Tavoian, E. Fiona Bailey, Janet L. Funk, Dawn K. Coletta

**Affiliations:** ^1^ Department of Physiology, University of Arizona, Tucson, AZ, United States; ^2^ Department of Medicine, Division of Endocrinology, University of Arizona, Tucson, AZ, United States; ^3^ Center for Disparities in Diabetes, Obesity and Metabolism, University of Arizona, Tucson, AZ, United States

**Keywords:** inspiratory muscle strength training, type 2 diabetes mellitus, glycemic control, insulin sensitivity, endothelial function

## Abstract

Type 2 diabetes mellitus (T2DM) is a complex, chronic metabolic disease that carries with it a high prevalence of comorbid conditions, making T2DM one of the leading causes of death in the U.S. Traditional lifestyle interventions (e.g., diet, exercise) can counter some adverse effects of T2DM, however, participation in these activities is low with reasons ranging from physical discomfort to lack of time. Thus, there is a critical need to develop novel management strategies that effectively reduce cardiometabolic disease risk and address barriers to adherence. High-resistance inspiratory muscle strength training (IMST) is a time-efficient and simple breathing exercise that significantly reduces systolic and diastolic BP and improves vascular endothelial function in adults with above-normal blood pressure. Herein we describe the study protocol for a randomized clinical trial to determine the effects of a 6-week IMST regimen on glycemic control and insulin sensitivity in adults with T2DM. Our primary outcome measures include fasting plasma glucose, fasting serum insulin, and insulin resistance utilizing homeostatic model assessment for insulin resistance (HOMA-IR). Secondary outcome measures include resting systolic BP and endothelium-dependent dilation. Further, we will collect plasma for exploratory proteomic analyses. This trial seeks to establish the cardiometabolic effects of 6 weeks of high-resistance IMST in patients with T2DM.

## Introduction

Type 2 diabetes mellitus (T2DM) is at epidemic proportions in the United States, affecting 38.4 million people (https://www.cdc.gov/diabetes/php/data-research/index.html). In 2022, the annual economic cost was $412.9 billion, making it the most expensive chronic condition in the U.S (1).. T2DM is a chronic, obesity-associated metabolic disorder characterized by glucose dysregulation, insulin resistance and beta-cell defects (2, 3). Adults with T2DM are likely to develop vascular endothelial dysfunction and vascular inflammation, increasing their risk of cardiovascular disease (CVD) and the occurrence of cardiac events (4). First-line T2DM treatments include lifestyle modifications, such as dietary changes and exercise (5), the benefits of which are well established in T2DM patients (6, 7).

According to the American Diabetes Association, individuals with T2DM should exercise at least 150-175 minutes per week, incorporating both aerobic and resistance-based activities (8). Although the benefits of exercise for T2DM are well-established, its prevalence continues to rise, likely due in part to inadequate adherence to recommended physical activity guidelines. Studies show that only 41% of adults with T2DM in the U.S. meet the current aerobic exercise guidelines, and only 12% meet the resistance exercise guidelines (9). In comparison, the general population has participation rates of 52% and 21%, respectively (9). Barriers such as physical discomfort and lack of time contribute to these low adherence rates (10, 11). Other factors affecting adherence include socioeconomic status, education level, health status, and physical fitness (12). Additionally, individuals with T2DM often experience reduced exercise tolerance due to early diabetes-related cardiopulmonary impairments, such as reductions in peak workload, peak oxygen uptake, and ventilatory efficiency, which can hinder the effectiveness of exercise training (13). Consequently, many individuals with T2DM remain sedentary, increasing their risk of developing other conditions and comorbidities (14, 15). Addressing and overcoming obstacles to exercise is essential because doing so can greatly enhance the health of patients with T2DM and other related conditions.

Recently, a novel, time-efficient respiratory exercise called Inspiratory Muscle Strength Training (IMST) was developed (16). IMST is distinct from other traditional forms of exercise due to its abbreviated training format (i.e., 5 minutes daily), and is performed using a hand-held device while seated or standing (16). With just six weeks of training (5 days/week), high resistance-IMST has been shown to lower systolic blood pressure (systolic BP) ~9 mmHg in normotensive and hypertensive adults (17). Furthermore, it has been shown to improve endothelium-dependent dilation (EDD) by 45% in older adults with elevated blood pressure (18). These vascular effects of IMST are believed to reduce the risks of CVD, the number one cause of death in people with T2DM (19). IMST is safe and well tolerated, with adherence rates >90% in diverse populations (18, 20), and thus presents a manageable introductory or adjunctive program for improving cardiometabolic health in T2DM patients who have difficulty maintaining a traditional exercise program. However, the effects of IMST on glycemic control and insulin sensitivity, as well as systolic BP and EDD are unknown in patients with T2DM.

Vascular endothelial function and metabolic function are closely linked (21). The vascular endothelium produces nitric oxide (NO), which is released in response to increased arterial wall shear stress (i.e., increased blood flow) (22). Among its various functions, NO enhances glucose uptake into cells and improves insulin sensitivity (23). T2DM is associated with impairments in endothelial function, including reduced NO production and increased vascular inflammation (24). High resistance-IMST is a potentially effective tool to combat T2DM-associated endothelial dysfunction, as it has been shown to increase NO bioavailability and reduce oxidative stress (18)— key adaptations that could improve metabolic health. The latter is especially significant given the link to metabolic syndrome, which encompasses insulin resistance, impaired glucose metabolism, and hypertension (25), and therefore, heightens the risks for cardiac event or stroke (25).

The potential for IMST to elicit cardiometabolic adaptations in diabetic patients warrants assessment. Accordingly, we outline a plan to interrogate the effects of 6 weeks of high-resistance IMST on glycemia (fasting plasma glucose), insulin sensitivity/resistance (fasting serum insulin and Homeostasis Model Assessment [HOMA-IR; ratio of fasting insulin/glucose]), resting BP, and NO-mediated EDD in T2DM patients. Participants will be randomized into either high-resistance (experimental) or low-resistance (control) groups, and complete IMST at home 5 days/week for 6 weeks, with each session lasting ~5 minutes (26). Participants will perform at either high relative resistance (75% of maximal inspiratory pressure in cmH_2_O, (PI_max_)) or low relative resistance (15% of PI_max_) (26). As this is a blinded study, the 15% will ensure participants feel a resistance, but there are not consistent cardiovascular improvements established in previous studies (17, 27, 28).

We will study T2DM patients before and after 6 weeks of high-resistance IMST to test the hypotheses that (1) fasting plasma glucose will decrease, and insulin sensitivity will improve, (2) resting systolic BP will decrease, and (3) high-resistance IMST will improve EDD resulting in clinically-meaningful improvements (i.e., >1% unit change) (29). We will obtain consent to collect blood DNA for banking and plasma for quantitative proteomics analysis. This will allow us to investigate novel protein expression changes before and after IMST. Omic technologies, such as proteomics, provide powerful tools for identifying biomarkers and developing new treatments (30, 31). Our study aims to use quantitative proteomics analyses to identify potential associations and putative markers related to the effects of IMST, which remain largely unexplored. We anticipate observing changes in protein abundance associated with metabolic and cellular processes following IMST, particularly in relation to outcomes in T2DM. However, it is important to note that this exploratory experiment focused on associations rather than mechanisms.

## Materials and methods

### Study design

**Figure 1:**
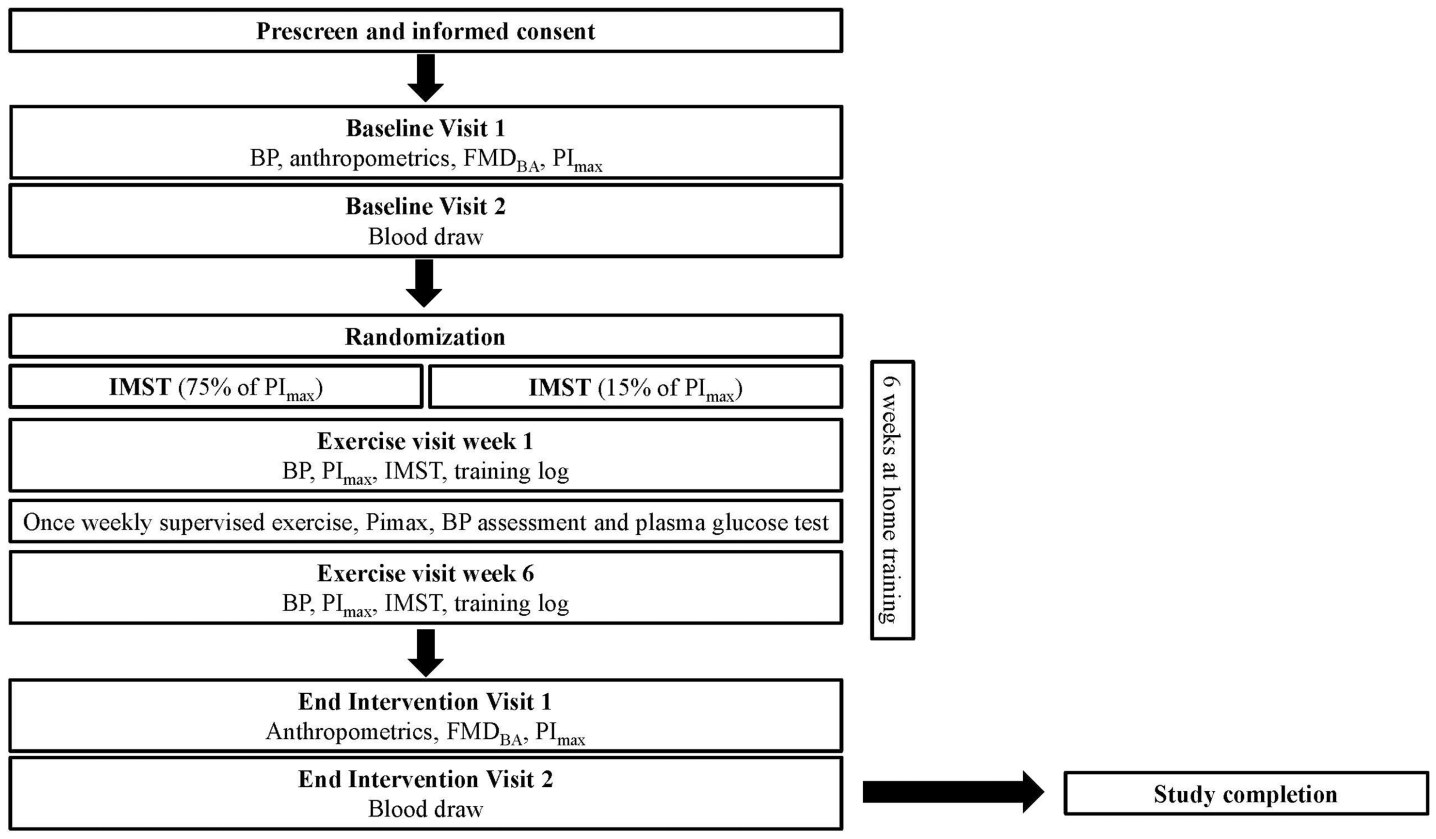
Study flow for the DIT study.

The Diabetes Inspiratory Training (DIT) study is a randomized, sham-controlled, exploratory clinical trial examining the effects of IMST in 24 adults with T2DM. This is a 6-week intervention study design. An outline of the study is shown in **Figure 1**.

Participants will perform 5 sets of 6 breaths per day, 5 days per week, at either high relative resistance (75% of maximal inspiratory pressure in cmH_2_O, (PI_max)_) or low relative resistance (15% of PI_max_) (26).

## Participants and interventions

### Study setting and study population

The DIT Study will be conducted at the Clinical and Translational Sciences (CATS) Research Center at the University of Arizona. Participants will be pre-screened over the phone to determine their eligibility using the inclusion and exclusion criteria (**Table 1**).

**Table 1:** Inclusion and exclusion criteria for DIT study.

Inclusions	Exclusions
18+ years old	Current smoker (tobacco/cannabis)
Diagnosed with type 2 diabetes by physician	Uncontrolled medical condition (e.g., cancer)
Fasting plasma glucose levels ≥126 mg/dl and ≤240 mg/dl	Myocardial infarction or stroke within the previous 12 months
SBP between 120-169 mmHg	Performs regular aerobic exercise (>4 bouts/week)
Stable dose of medication (3 months on same dose)	DBP >100 or <60 mmHg
Weight stable in the prior 3 months (< 3 kg weight change) and willing to remain weight stable throughout the study	SBP <120 or ≥170 mmHg
Absence of unstable clinical disease as determined by medical history	Medications that, in the opinion of the study physician or nurse practitioner, may impact the outcomes of the study (e.g., steroids)
	Cheyne-Stokes Respiration
	History or perforated eardrum
	History of glaucoma or retinopathy
	Pregnant, breastfeeding or trying to become pregnant (self-reported)

SBP, systolic blood pressure; DBP, diastolic blood pressure; mmHg, millimeters of mercury; mg/dl, milligrams per deciliter; kg, kilograms; kg/m^2^, kilograms per meter squared.

### Eligibility criteria

Participants will be eligible for this study if they 1) are 18 years of age or older, 2) have been previously diagnosed with T2DM by a physician, 3) have a systolic BP between 120-169 mmHg, 4) are on a stable dose of medication for at least 3 months, 5) do not have an unstable clinical disease, and 6) do not meet the exclusion criteria (**Table 1**). The participants will be asked to maintain their current diet and to not begin any new diet/weight management strategies until their participation in the study is over. We will encourage the participants to eat a balanced meal the day before their scheduled study visit.

### Interventions

All training will be completed using the POWERbreathe™ K3 trainer (POWERbreathe International Ltd., Warwickshire, U.K.). This is a handheld pressure-threshold device with a computerized threshold sensor. Each participant will be provided with their own device on which they will perform IMST at home, 5 days/week for 6 weeks. They will receive in-person verbal instruction on the training protocol and K3 operation from the Research Technician at the start of the study.

The Research Technician will monitor one training session each week in the CATS facility; the remaining 4 sessions will be completed unsupervised at home. During each visit to the CATS facility, the Research Technician will determine the participant’s PImax and transfer the saved training data from the K3 device to ensure exercises are being completed at home. The PImax will be determined by taking the average of 3 measurements. The Research Technician will then adjust the training resistance as needed to ensure participants are training at the prescribed intensity (i.e., either 15% or 75% of PImax). The participant will then perform a supervised training session. Adherence will be assessed by comparing the quantity and quality of completed training sessions to those prescribed. Monitoring will be conducted using the internal data storage of the POWERbreath™ K3 device. Training session data will be downloaded weekly, prior to supervised sessions, to evaluate participant adherence for the preceding week. Participants also will be required to complete a weekly training log to track any additional exercises outside of the daily IMST training. The safety of the training intervention will be assessed by recording any adverse events reported by the participants. Tolerability will be measured by the rate at which enrolled participants dropped out due to adverse events.

## Outcomes

### Primary outcomes

#### Fasting plasma glucose and fasting serum insulin

Participants will report to the CATS facility at the University of Arizona following a 12-hour fast for both the baseline visit and post-intervention visit. Up to 5 mL of blood will be drawn from the antecubital vein and sent to Sonora Quest for screening laboratory tests, lipid measures, and metabolic panels, including fasting plasma glucose and fasting serum insulin.

#### Insulin sensitivity

Insulin sensitivity will be assessed using the Homeostatic Model Assessment for Insulin Resistance (HOMA-IR), a validated surrogate method for estimating insulin sensitivity (32, 33). The equation for HOMA-IR is:


$$HOMA-IR=\frac{\left(fasting\;serum\;insulin\left(\frac{\mu IU}{mL}\right)\right)x\left(fasting\;plasma\;glucose\left(\frac{mg}{dL}\right)\right)}{405}$$

### Secondary outcomes

#### Resting blood pressure

We will measure resting blood pressure per the American College of Cardiology (ACC) and the American Heart Association (AHA) guidelines (26) with an automated oscillometric sphygmomanometer (SunTech CT40, SunTech Medical). Briefly, participants will be asked to sit quietly with both feet flat on the ground, backs supported, and with their arms resting at heart level (26). Three measures will be performed after a 5-minute quiet rest period with 1 minute of recovery between each measure. The average systolic and diastolic blood pressures will be recorded at pre- and post-intervention.

#### Endothelium dependent dilation

Endothelium Dependent Dilation (EDD) will be assessed via brachial artery flow-mediated dilation (FMD) using high-resolution ultrasonography (Canon Xario 200G), as previously described (34). Participants will be asked to avoid exercise, caffeine, and alcohol for 24 hours, and food for at least 5 hours prior to their visit. FMD will be assessed by measuring the brachial artery diameter and blood velocity at baseline and for 3 minutes following reactive hyperemia which stimulates NO release. Reactive hyperemia is induced by 5 minutes of forearm blood flow occlusion with a cuff placed on the upper forearm and inflated at least 50 mmHg above systolic BP (26, 35). Brachial artery diameter and blood velocity will be analyzed offline using commercially available software (Brachial Analyzer, Medical Imaging Applications LLC, Coralville, IA, USA) (26) and expressed as absolute (mm) and percent change in arterial diameter from baseline (pre-cuff inflation diameter) to post-intervention following the 6 weeks of IMST.

### Exploratory outcomes

#### Proteomic analysis

Blood will be collected into purple K2-EDTA vacutainers and immediately placed on ice, then centrifuged at 3,000 rpm at 4°C within 10 minutes of blood collection. Separated plasma will be removed and frozen at -80°C in cryotubes until analyzed using high-performance liquid chromatograph-electrospray ionization-MS/MS (LC-MS) (36). Briefly, the extracted plasma proteins will be subjected to subsequent in-solution digestion using trypsin and Lys-C to be analyzed with tandem mass spectrometry (36, 37). Lastly, quantitative proteomics will be performed using extracted ion abundance, including statistical analysis via Progenesis (38). The resulting quantitative proteomic data sets will be analyzed using DAVID for gene ontology and pathway enrichment analysis (39, 40).

#### DNA banking

Blood will be collected from the antecubital vein of the arm directly into PAXgene DNA collection tubes, as per manufacturer’s instructions. Briefly, these tubes contain an additive reagent that stabilizes the blood. The tubes will sit at room temperature for 2 hours then will be stored in the -20 freezer until ready to be processed. The PAXgene DNA processing kit will be used for isolation of the DNA. Once DNA is extracted, it will be stored and banked for future studies. Participants will be required to provide their consent for banking of their de-identified DNA/plasma samples.

### Participant timeline

The timeline for participation in the study will be 7-9 weeks as shown in **Figure 1**. A summary of the visits for the DIT study participants is shown in **Table 2**.

**Table 2:** Summary of the visits for the DIT study.

	Baseline		Training	End-Intervention	
	Visit 1	Visit 2	Week 1-6	Visit 1	Visit 2
Screening					
Informed Consent	X				
Demographics	X				
Medical History	X				
Inclusion/Exclusion	X				
Hip/waist/neck circumference	X			X	
Functional Assessments					
Resting BP	X		X	X	
PI_max_	X		X	X	
Spirometry	X			X	
Blood Draw					
Metabolic panel		X			X
Plasma/DNA		X			X
Arterial Assessments					
FMD_BA_	X			X	
At-home Testing					
Sleep/exercise diary			X		
Exercise sessions					
Daily exercise			X		
Once-weekly supervised exercise session*			X		
Weekly text/email			X		
Randomization		X			

BP, Blood Pressure; FMD_BA_, Flow-mediated dilation of brachial artery; PI_max_, Maximal inspiratory mouth pressure. *, At this weekly supervised training session, the participant will do a fasting plasma glucose check immediately before and after the training session.

Participants will sign a written informed consent with a member of the research team at the CATS facility. Following informed consent, participants will complete all baseline assessments in two in-person visits to the CATS facility within a 14-day window. Participants will begin the 6-week intervention ≤ 14 days after baseline assessments are completed. If the participant is unable to make one of their in-person training sessions, they will proceed to do their exercises at home and come the following week for their regularly scheduled training session in order to maintain the 6-week training regimen. During the weekly training session at the CATS facility, a weekly blood pressure check will be performed (41). In addition, during the weekly visit, plasma glucose levels will be measured immediately before and after the exercise session. These measurements are exploratory and aim to identify any immediate effects of IMST on plasma glucose levels. All assessments will be repeated within 14 days after completion of the 6-week training program. Participants will continue to perform IMST 5 days/week until all post assessments are completed.

### Power analysis and sample size

A minimum of 16 and a maximum of 24 participants will be enrolled and randomized into groups. To our knowledge, the effect of IMST on fasting glucose and/or insulin sensitivity in any population have not previously been reported, nor have effects of IMST on systolic BP and EDD in T2DM been specifically ascertained. Thus, for our power analysis we estimated a modest effect size of 0.40 with alpha set at 0.05 using a repeated measures ANOVA within-between framework. A sample size of 16-24 will have 85-96% power for any outcome with an effect size of ≥ 0.40.

### Recruitment

We will strive to maintain a 1:1 male-to-female ratio within the study groups. Recruitment will be via word of mouth, advertisements placed in area newspapers, social media, and flyers posted around the University of Arizona and to the surrounding local community in Tucson. Interested individuals will be directed to the study website where they will be able to complete a questionnaire to determine their eligibility. Individuals who do not meet the inclusion criteria will be informed of their ineligibility. Candidates that meet eligibility will be contacted for a study overview session.

After the study overview, written informed consent will be obtained in person from each participant before the start of any study-related procedures. Ethical Approval for this study has been obtained from the University of Arizona Institutional Review Board (Protocol 00002239).

## Assignment of interventions

### Sequence generation

The randomization sequence will be created using computer-generated random numbers at a 1:1 ratio in blocks of four. Male and female participants will be randomized using separate randomization tables.

### Allocation concealment mechanism

Group allocation will be stored in an Excel file that is not available to the Research Technician.

### Implementation

Once the Research Technician has completed all enrollment activities for a participant (i.e., a participant has met the inclusion criteria and completed baseline assessments), the Principal Investigator (PI) will inform the research technician of the participant’s allocation group.

### Blinding

Due to the nature of the study, the participants are blinded to the intervention.

## Data collection, management, and analysis

### Data management

Data will be collected with paper data collection forms and entered into a Microsoft Excel sheet within 48 hours of data collection. At the end of the study the Excel sheet will be rechecked against the paper originals and any inconsistencies will be noted and discussed between the PI and Research Technician in charge of data entry.

### Statistical plan

Data will be analyzed with a repeated measures ANOVA test and Sidak *post hoc* testing using SPSS version 28.0. We will examine both group-by-sex and group-by-age interactions, and report effect sizes along with confidence intervals, in addition to p-values. All tests will be two-sided with alpha set at 0.05.

## Monitoring

### Data monitoring

The intervention is low-risk and does not require a data monitoring committee. The research team will meet with the study physician at regular intervals to track study progress and discuss any potential safety issues. No interim analyses will be performed.

### Harms

An adverse event (AE) is any harmful and unintended reaction during the course of the study that may be related or unrelated to the intervention. All AEs occurring between a participant signing the informed consent and completing post-intervention assessments will be reported to the study physician.

## Anticipated results

### Primary hypothesis

Six weeks of high-resistance IMST will lower fasting plasma glucose and improve insulin sensitivity.

### Other hypotheses

Six weeks of high-resistance IMST will:

1. Lower resting systolic BP
2. Improve EDD

## Discussion

Regular exercise is one of the most commonly prescribed non-pharmacological interventions for T2DM management and yields improvements in glycemic control and insulin action (6, 7). However, aerobic exercise is physically strenuous and time-consuming (10, 11) and less than half of T2DM adults participate in exercise on a regular basis. IIn contrast, IMST is a novel form of high intensity training that can be performed whether sitting or standing requires only 5 minutes per day, and rapidly improves blood pressure, endothelial vascular function, and vascular resistance among hypertensive adults (17, 18, 20, 28). Whether IMST can also affect changes in fasting blood glucose or insulin sensitivity is of critical interest and important for adults with T2DM, along with establishing if these blood pressure lowering effects and increased EDD are also seen in this population following IMST.

A study by Corrêa et al. studied the acute effects of IMST on glucose variability and showed significant improvements in glucose immediately following the training (42). Additionally, another study which was for 12 weeks at a lower resistance of 30% revealed no significant changes in blood glucose levels (43). The discrepancies across these findings are likely due to populations studied, the timeframe of the training and the resistance used. To our knowledge there have been no investigations that have reported the effects of chronic IMST training at a resistance of 75% or an interval training protocol similar to ours on glycemic control and insulin sensitivity in T2DM.

**Figure 2:**
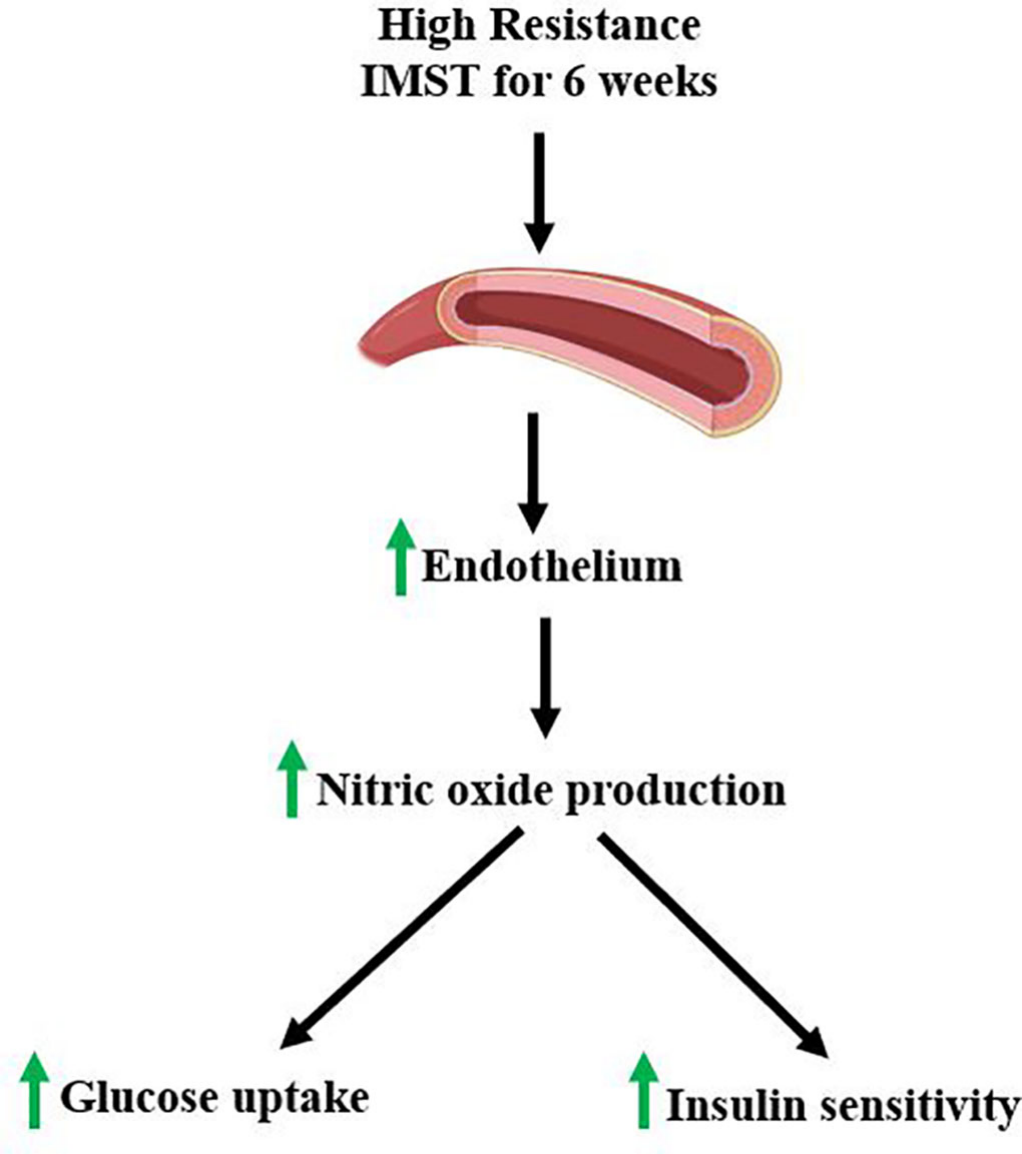
IMST proposed mechanism of action.

We expect the IMST intervention to enhance vascular health, as well as improvements in metabolic health. We propose that IMST may enhance insulin sensitivity and endothelial function by increasing NO bioavailability and reducing oxidative stress (**Figure 2**). Previous research by Bailey et al. demonstrated that endothelial function and NO bioavailability improved after six weeks of high-resistance IMST in older adults with normal to elevated blood pressure (18). This suggests that IMST enhances endothelial function by activating endothelial nitric oxide synthase (eNOS), which likely increases NO levels essential for vasodilation and improved blood flow (18). This mechanism reduces ROS, maintains NO levels, and supports vascular health. The increased NO not only improves endothelial function but also enhances glucose uptake by improving blood flow to skeletal muscles (23), potentially boosting insulin sensitivity. We hypothesize that these pathways activated by IMST could improve cardiometabolic health, particularly in individuals with T2DM.

To conclude, high-resistance IMST has the potential to offer long-term benefits for patients with T2DM, similar to those observed in other populations (16, 18, 20, 26, 29). High adherence rates are anticipated, positioning IMST as a valuable first-line treatment option for T2DM. Additionally, it could serve as a preparatory tool for engaging in traditional aerobic or resistance exercise programs. Expected physiological improvements include reduced fasting plasma glucose levels, enhanced insulin sensitivity, lower systolic blood pressure, and improved endothelial function.

## Ethics and dissemination

### Research ethics approval

This study has been approved by the University of Arizona Institutional Review Board (Approval Number: 00002239).

### Protocol amendments

Any modifications to the protocol that may impact the conduct of the study will first be decided by the PI and approved by the University of Arizona IRB prior to any implementation. Administrative changes of the protocol are considered minor corrections that have no impact on the way the study is to be conducted. These changes will be agreed upon by the PI and documented.

### Consent

This study will be thoroughly explained to each participant in person where subjects will have the opportunity to ask questions. Once the member of the research team believes the participant understands the study requirements, they will be directed to read and sign the informed consent document.

### Confidentiality

The identity of the participants will be protected by assigning each a code (i.e., a 3-digit number) and any experimental data collected from these subjects will be recorded under that number. Any identifiable personal information will be kept in a password-protected digital file and/or in a locked cabinet. Only the PI, Co-I and Research Technician will have access to the information.

### Access to data

The PI, Co-I and Research Technician will have access to the final trial dataset. Other project team members will be provided with de-identified data for their analysis.

### Dissemination policy

Primary outcome papers will be approved by the PI prior to journal submission. Every attempt will be made to release study results to the general public soon after study completion. Interim and final reports may also be presented at various local, regional, and international conferences, with approval from the PI. Eligibility for authorship include (1) substantial contribution to study conception and design AND/OR substantial contributions to acquisition analysis or interpretation of data, AND (2) drafting or revising the manuscript, AND (3) final approval of the manuscript. There is no intention to use professional writers.
